# The effect of quail egg supplements enriched with marine macroalgae *Eucheuma* spinosum on the physiological condition of Sprague Dawley rats during pregnancy

**DOI:** 10.5455/javar.2024.k862

**Published:** 2024-12-29

**Authors:** Hasan Basri, Slamet Widiyanto, Hendry T. S. Saragih, Zuprizal Zuprizal

**Affiliations:** 1 Biology Doctoral Study Program, Faculty of Biology, Universitas Gadjah Mada, Yogyakarta, Indonesia; 2 Biology Study Program, Faculty of Mathematics and Natural Science, Universitas Islam Al-Azhar, Mataram, Indonesia; 3 Department of Animal Physiology, Faculty of Biology, Universitas Gadjah Mada, Yogyakarta, Indonesia; 4 Laboratory of Animal Development Structure, Faculty of Biology, Universitas Gadjah Mada, Yogyakarta, Indonesia; 5 Department of Animal Nutrition and Feed Science, Faculty of Animal Science, Universitas Gadjah Mada, Yogyakarta, Indonesia

**Keywords:** Quail egg, marine macroalgae, blood cell, lipid, pregnancy period

## Abstract

**Objective:**

To investigate the effect of quail egg supplements enriched with marine macroalgae *Eucheuma spinosum* on body weight and physiological conditions of *Sprague Dawley* rats during pregnancy.

**Materials and Methods:**

This study used a completely randomized experimental design. The test animals were 25 pregnant white rats aged 3 months and weighed ± 200 gm. Pregnant rats were divided into five treatments and five repetitions; each repetition contained one pregnant rat. T0: control treatment; T1: treatment group consuming quail eggs from quail fed commercial feed; T2: treatment group consuming quail eggs from quail fed with 3% *E. spinosum*; T3: treatment group consuming quail eggs from quail fed with 4% *E. spinosum*; T4: treatment group consuming quail eggs from quail fed with 5% *E. spinosum*. The parameters measured were egg proximate, egg iron, egg cholesterol, red blood cell (RBC), hemoglobin (Hb), hematocrit (HCT), mean corpuscular volume (MCV), mean corpuscular Hb (MCH), mean corpuscular Hb concentration (MCHC), white blood cell (WBC), lymphocytes (LYM), *neutrophils* (NEUT), RBC distribution width (RDW), platelet distribution width (PDW), mean platelet volume (MPV), cholesterol, high-density lipoprotein (HDL), low-density lipoprotein (LDL), glucose, *superoxide dismutase* (SOD), and m*alondialdehyde* (MDA).

**Results:**

The addition of *E. spinosum* into quail feed at concentrations of 3%, 4%, and 5% did not show statistically significant differences (*p *> 0.05) in the proximate levels (water, ash, fat, protein, and carbohydrates) compared to the control. However, the analysis of iron in quail eggs indicated a significant increase (*p *< 0.05) in comparison to the control treatment. In addition, the supplementation of *E. spinosum* in the quail feed resulted in significantly reduced (*p *< 0.05) quail egg cholesterol levels when compared to the control treatment. Furthermore, the supplementation of quail egg produced by quail fed with *E. spinosum* did not significantly affect the birth weight of the pups, RBC, MCV, MCH, MCHC, WBC, LYM, NEUT, RDW, PDW, MPV, MDA, and SOD when compared to the control treatment (*p* > 0.05). Meanwhile, the mother weight, Hb, HCT, cholesterol, HDL, LDL, and glucose levels significantly increased (*p* < 0.05) compared to the control.

**Conclusion:**

The administration of *E. spinosum *with concentrations of 3%, 4%, and 5% can increase iron levels and decrease cholesterol levels in quail eggs. The administration of quail egg supplements produced by quail-fed additional macroalgae *E. spinosum* can increase body weight, Hb, and HCT in pregnant rats. The addition of marine macroalgae with concentrations of 4% and 5% can decrease the levels of cholesterol, LDL, and glucose and increase serum HDL levels in pregnant rats.

## Introduction

Pregnancy is a period when the mother undergoes significant physiological changes, beginning with fertilization and continuing until birth. The US Dietary Guidelines (2020–2025) highlight pregnancy as a special stage in life. Nutrition plays a vital part in the pregnancy process, starting before, during, and after pregnancy to support the health of the mother and child [1]. There are increases in nutritional needs during pregnancy compared to normal conditions that are used for fetal and placental growth, increased blood volume, mammary gland development in preparation for breastfeeding, and an increase in body metabolism [2].

Pregnancy is the period most vulnerable to physiological disorders. Physiological disorders in pregnancy include oxidative stress and anemia. An imbalance between oxidants and antioxidants leads to oxidative stress, with oxidant levels being more prevalent. This imbalance can cause fetal growth disorders, premature birth, and low birth weight. Meanwhile, anemia during pregnancy is a maternal ailment with levels of hemoglobin (Hb) in the blood <11.0 gm/dl or a condition in which the number of erythrocytes is lower than normal due to a deficit of one or more blood cell formation components [3]. Most anemia that occurs during pregnancy is caused by nutritional factors, acute bleeding, and low iron intake. In this period, the need for iron increases compared to normal conditions. During pregnancy, insufficient iron levels elevate the health risks for both the mother and the developing fetus. Anemia during pregnancy is strongly linked to low birth weight, fetal death, miscarriage, growth retardation, lower iron reserves in children, and children born with nutritional anemia [4].

During pregnancy, various micronutrients are required, including folic acid, calcium, iron, zinc, iodine, and vitamins. These nutritional demands can be obtained from food intake. Consuming quail eggs during pregnancy can provide essential nutrients as an alternative dietary option. Quail eggs are an inexpensive yet extremely nutritious food that delivers balanced nutrients with an impact on health. Quail eggs contain fat, protein, carbohydrates, fiber, essential fatty acids, vitamins, minerals, lipids, and bioactive compounds with antioxidant properties. The nutritional content of quail eggs can be enhanced using feed additives. Marine macroalgae can be utilized as a feed additive. Previous studies showed that marine macroalgae flour (*Macrocystis pyrifera*) as a feed additive can improve the nutritional content of chicken eggs [5]. Appropriate administration of Fe concentrations can increase iron concentrations in yolk and egg albumen [6].

Despite the potential health advantages, quail eggs are not commonly used as a nutritional source for pregnancy. A previous study has shown that consuming one boiled chicken egg per day for a week can effectively boost Hb levels in pregnant women [7]. In addition, supplementing eggs to pregnant rats has been found to have notable changes in body weight, length, and mass index [8]. In another study, chicken eggs given to pregnant rats resulted in normal birth weights and glucose levels similar to the control treatment [9]. These findings underscore the vital role of egg consumption during pregnancy and suggest that it should be encouraged as a dietary supplement for optimal maternal and fetal wellness.

Limited research has been conducted on the effects of quail eggs as a nutritional intake during pregnancy, with previous studies only utilizing chicken eggs [7]. There is currently no existing research on the effects of quail egg supplementation on the morphological and physiological conditions of pregnancy. Therefore, the purpose of this study is to investigate the impact of quail egg supplements enriched with marine macroalgae (*E. spinosum*) on blood cells, lipids, blood glucose levels, and antioxidant activity in pregnant rats. The results of this study offer valuable insight into the potential implications of quail egg supplementation for pregnancy outcomes and may have implications for future research in this area.

## Materials and Methods

This study was conducted in the Laboratory of the Faculty of Animal Husbandry, Integrated Laboratory for Researching and Testing (LPPT), and Laboratory of Food and Nutrition, Universitas Gadjah Mada. This study was conducted after obtaining approval from the Ethical Clearance Commission with Certificate Number: 00026/04/LPPT/VIII/2023.

This study used female Sprague-Dawley rats. The animal subjects were kept in a controlled environment with the temperature maintained at 25°C and 70% humidity. The rats were subjected to a 12-h cycle of light and dark. The rats were given food and water ad libitum, with food given for 15-h during the night (17:00–07:00) and 9-h during the day (08:00–16:00). They were given standard feed (Rat Bio) from PT. Citra Ina Feedmill, which contained 60% carbohydrates, 20% protein, 4% fat, 4% crude fiber, 12% calcium, and 0.7% phosphorus.

The determination of the estrous cycle is carried out by the vaginal smear method. The first mating process was initiated by placing three female rats that had gone through the estrous phase and one male rat in a cage sized 37 × 30 × 20 cm. A vaginal smear was carried out every morning to verify if the mating has occurred. This is indicated by the presence of cells during estrus and dead sperm.

The administering treatment is by boiling the quail eggs for 2 min, cutting them into small pieces, and feeding them to the rats daily at 4:00 pm.

This is an experimental study with a completely randomized design. A total of 25 pregnant white rats (*R. norvegicus* L.) aged 3 months and weighing approximately 200 gm were used as the test animals. The test animals in each treatment group were placed randomly. The pregnant rats were divided into five treatment groups, namely:

T0: control treatment;

T1: treatment group consuming quail eggs from quail fed commercial feed;

T2: treatment group consuming quail eggs from quail fed with 3% *E. spinosum*;

T3: treatment group consuming quail eggs from quail fed with 4% *E. spinosum*;

T4: treatment group consuming quail eggs from quail fed with 5% *E. spinosum*.

Each treatment group consisted of five replications. Each replication consisted of one pregnant rat (*R. norvegicus L*.).

Blood samples were collected on the 21st day of pregnancy from the orbital sinus of the eye. Before the blood sampling, anesthesia was administered using a combination of ketamine (50–75 mg/kg) and xylazine (30–40 mg/kg). The body weight of the mother and rat pups was measured immediately following birth using a digital scale accurate to 0.01 gm and was calibrated.

Complete blood analysis (red blood cell (RBC), Hb, hematocrit (HCT), mean corpuscular volume (MCV), mean corpuscular Hb (MCH), white blood cell (WBC), lymphocytes (LYM), *neutrophils* (NEUT), RBC distribution width (RDW), platelet distribution width (PDW), and mean platelet volume (MPV)) was carried out using the hematology analyzer method [10]. Analysis of cholesterol, high-density lipoprotein (HDL), and low-density lipoprotein (LDL) levels using the cholesterol oxidation-phenol-4-aminoantipyrine-peroxine method; blood glucose using Glucose Oxsidase–Aminoantypyrine Peroxidase method using Ultraviolet-visible spectrophotometry [11]. Analysis of *superoxide dismutase* (SOD) and *malondialdehyde* (MDA) using an Enzyme-Linked Immunosorbent Assay kit from Bioassay Technology Laboratory (Shanghai, China).

The data were analyzed using ANOVA, and if a significant difference was found, the Duncan test was performed at a 95% significance level using a statistical package for the social sciences 22 for Windows software.

## Results

### Nutrition and cholesterol content in quail eggs

The nutritional composition of quail eggs, including proximate (water, ash, fat, protein, and carbohydrate), iron, and cholesterol, is presented in Table 1. Results from the addition of *E. spinosum* at certain concentrations (3%, 4%, and 5%) did not show significant differences (*p* > 0.05) in the water, ash, fat, protein, and carbohydrate content compared to the control treatment. However, the analysis revealed a significant increase in iron levels (*p* < 0.05) compared to the control treatment. The highest iron level in T2 treatment (31.30 mg/100 gm). In addition, the analysis also showed that the cholesterol level significantly decreased (*p *< 0.05) compared to the control, with the lowest cholesterol levels observed in the T3 treatment with an additional 5% *E. spinosum.*

### The body weight of mother rats and rat pups’ birth weight

Table 2 presents the data on the mother’s weight and the birth weight of the rat pups. The supplementation of quail eggs produced by quail fed additional feed with *E. spinosum* significantly increased (*p *< 0.05) the weight of the mother rats compared to the control treatment. The highest weight of the mother rat was in treatment T4 (280.53 gm). However, the analysis of the birth weight of rat pups revealed no significant difference (*p *> 0.05) compared to the control treatment.

### Blood cells of rats during pregnancy

Data regarding the levels of RBC, Hb, HCT, MCV, MCH, mean corpuscular Hb concentration (MCHC), WBC, LYM, NEUT, RDW, PDW, and MPV are in Table 3. The results of the study indicated that the supplementation of quail eggs produced by quails fed with additional feed containing *E. spinosum* statistically showed no significant effect (*p *> 0.05) on RBC levels, MCV, MCH, MCHC, WBC, LYM, NEUT, RDW, PDW, and MPV compared to the control. However, the Hb and HCT levels in the treatment group showed significant increases (*p *< 0.05) compared to the control. The highest Hb levels were found in T2 (12.13 gm/dl) and HCT T3 (33.08%).

**Table 1.** Nutrient content of quail eggs after being given additional feed with marine macroalgae (*E. spinosum*).

Parameters	T0	T1	T2	T3
Water (%)	71.63 ± 0.72	72.69 ± 0.55	72.54 ± 0.51	72.73 ± 0.34
Ash (%)	0.92 ± 0.03	0.93 ± 0.03	0.93 ± 0.02	0.95 ± 0.04
Fat (%)	10.71 ± 0.32	10.42 ± 0.58	10.32 ± 0.61	10.12 ± 0.33
Protein (%)	12.68 ± 0.19	12.79 ± 0.35	12.46 ± 0.35	12.95 ± 0.17
Carbohydrates (%)	3.32 ± 0.23	3.34 ± 0.35	3.61 ± 0.41	3.14 ± 0.70
Iron (mg/100g)	27.01 ± 0.47^a^	30.62 ± 0.93^c^	31.30 ± 1.28^c^	29.38 ± 0.44^b^
Cholesterol (mg/100g)	854.97 ± 37.13^a^	573.60 ± 56.47^b^	439.77 ± 23.15^c^	364.14 ± 34.86^d^

Note: Different superscripts indicate there is a significant difference (*p *< 0.05). T0: fed commercial feed; T1: fed *E. spinosum *3%; T2: fed 4% *E. spinosum; *T3: fed *E. spinosum *5%.

**Table 2.** Mother’s body weight and birth weight of rat offspring after being given quail egg supplements.

Parameters	T0	T1	T2	T3	T4
Initial weight (gm)	172.82 ± 3.89	182.19 ± 11.39	190.35 ± 23.08	189.55 ± 9.19	200.93 ± 10.36
Final weight (gm)	233.37 ± 26.07^a^	263.39 ± 16.12^ab^	261.94 ± 30.42^ab^	279.61 ± 20.06^b^	280.53 ± 13.19^b^
Overall weight gain (gm)	60.55	81.20	71.59	90.06	79.60
Daily weight gain (gm)	2.88	3.87	3.41	4.29	3.79
Birth weight of rat pups	5.47 ± 0.24	6.26 ± 1.03	5.98 ± 0.78	6.29 ± 0.85	6.15 ± 0.62

Note: Different superscripts indicate there is a significant difference (*p* < 0.05). T0: control treatment; T1: treatment group given quail egg supplements produced by quail fed commercial feed; T2: treatment group supplemented with quail eggs produced by quail fed with 3% marine macroalgae *E. spinosum*; T3: treatment group supplemented with quail eggs produced by quail fed with 4% marine macroalgae *E. spinosum*; T4: treatment group that was supplemented with quail eggs produced by quail fed with 5% marine macroalgae *E. spinosum*.

**Table 3.** Hematological profile of pregnant mice after being given quail egg supplements.

Parameters	T0	T1	T2	T3	T4
**RBC** (×10^6^/µl)	5.45 ± 0.18	5.92 ± 0.19	6.13 ± 0.44	5.81 ± 0.31	6.01 ± 0.42
**Hb** (g/dl)	10.40 ± 0.29^a^	11.60 ± 0.18^b^	12.13 ± 0.81^b^	11.70 ± 0.80^b^	11.53 ± 0.92^b^
**HCT** (%)	29.18 ± 0.59^a^	32.40 ± 0.88^b^	32.36 ± 2.74^b^	33.08 ± 1.87^b^	31.95 ± 1.27^b^
**MCV** (fl)	54.63 ± 1.38	56.73 ± 0.70	56.93 ± 1.78	56.90 ± 0.37	56.63 ± 1.44
**MCH** (pg)	19.20 ± 0.74	20.25 ± 0.49	20.40 ± 0.94	20.13 ± 0.45	19.78 ± 0.44
**MCHC** (g/dl)	35.62 ± 0.90	35.74 ± 0.58	35.66 ± 0.67	35.38 ± 0.53	34.96 ± 0.83
WBC ×10^3^/µl	6.62 ± 0.45	8.34 ± 1.29	7.98 ± 1.52	7.82 ± 1.61	6.84 ± 2.17
**LYM** (×10^3^/µl)	4.12 ± 0.89	5.16 ± 1.41	5.14 ± 1.36	5.04 ± 1.34	3.64 ± 1.14
**NEUT** (×10^3^/µl)	2.98 ± 0.52	3.18 ± 0.44	2.84 ± 0.38	2.78 ± 0.33	3.20 ± 2.14
**RDW** (fl)	27.06 ± 1.22	26.84 ± 0.55	28.42 ± 1.45	27.72 ± 0.69	27.90 ± 0.69
**PDW** (fl)	7.58 ± 1.13	6.76 ± 0.40	6.48 ± 0.13	6.96 ± 0.49	5.54 ± 3.06
**MPV**(fl)	6.56 ± 0.58	6.00 ± 0.27	5.76 ± 0.11	6.12 ± 0.29	5.98 ± 0.47

Note: Different superscripts indicate there is a significant difference (*p* < 0.05). T0: control treatment; T1: treatment group given quail egg supplements produced by quail fed commercial feed; T2: treatment group supplemented with quail eggs produced by quail fed with 3% marine macroalgae *E. spinosum*; T3: treatment group supplemented with quail eggs produced by quail fed with 4% marine macroalgae *E. spinosum*; T4: treatment group that was supplemented with quail eggs produced by quail fed with 5% marine macroalgae *E. spinosum*.

### Serum biochemistry and the status of oxidative stress

The data in Table 4 presents the outcomes of the study on the effects of supplementing quail eggs produced by quails given additional feed with *E. spinosum* on cholesterol, HDL, LDL, glucose, SOD, and MDA levels. According to the study results, the supplementation led to an increase in cholesterol, HDL, LDL, and glucose levels (*p *< 0.05) compared to the control. The MDA and SOD analysis did not indicate any significant differences (*p *> 0.05) compared to the control treatment.

## Discussion

The addition of marine macroalgae as a supplement in quail feed can increase the iron content and decrease cholesterol levels in quail eggs. Seaweed has a high iron content of 118.71 mg/100 gm, which results in an increase in iron content in quail eggs. Previous research indicates that increasing dietary iron can elevate iron levels in both the yolk and albumen of chicken eggs [6]. Studies have shown that supplementing chicken diets with Fe-Gly at a dosage of 60 mg/kg significantly increases the iron content in egg yolk and albumen [12]. The transport of iron into quail eggs occurs through its binding to vitellogenin (VTG), a precursor of egg yolk protein synthesized by the liver in response to estrogenic stimulation. During the process of egg production, the liver synthesizes apolipoprotein and VTG, which are the primary components of egg yolk protein. VTG, a glycophospholipoprotein, is responsible for providing iron and is released into the bloodstream for transport to the oocyte. The addition of marine macroalgae feed at varying concentrations of 3%, 4%, and 5% resulted in a linear reduction in quail egg cholesterol levels, as shown in Table 1.

**Table 4.** Serum biochemistry and oxidative stress status in pregnant mice after being given quail egg supplements.

Parameters	T0	T1	T2	T3	T4
Cholesterol (mg/dl)	99.73 ± 0.51^a^	134.00 ± 6.36^b^	100.63 ± 8.72^a^	131.13 ± 0.93^b^	109.63 ± 11.20^a^
**HDL** (mg/dl)	37.70 ± 2.40^ab^	33.57 ± 7.70^a^	42.17 ± 5.58^ab^	56.13 ± 1.40^c^	45.47 ± 6.35^b^
**LDL** (mg/dl)	67.17 ± 1.00^a^	93.30 ± 2.65^c^	71.90 ± 5.90^ab^	91.70 ± 0.89^c^	77.43 ± 6.96^b^
Glucose (mg/dl)	23.80 ± 8.36^a^	58.47 ± 7.38^b^	55.03 ± 3.70^b^	50.67 ± 21.16^b^	41.83 ± 11.38^ab^
**MDA** (nmol/ml)	1.39 ± 0.07	1.28 ± 0.25	1.28 ± 0.05	1.28 ± 0.11	1.26 ± 0.025
**SOD** (ng/ml)	2.22 ± 0.24	1.92 ± 0.16	2.02 ± 0.19	2.37 ± 0.14	2.18 ± 0.15

Note: Different superscripts indicate there is a significant difference (*p* < 0.05). T0: control treatment; T1: treatment group given quail egg supplements produced by quail fed commercial feed; T2: treatment group supplemented with quail eggs produced by quail fed with 3% marine macroalgae *E. spinosum*; T3: treatment group supplemented with quail eggs produced by quail fed with 4% marine macroalgae *E. spinosum*; T4: treatment group that was supplemented with quail eggs produced by quail fed with 5% marine macroalgae *E. spinosum*.

The high antioxidant content (52.10%) in marine macroalgae plays an important role in reducing cholesterol levels in quail eggs. Previous study has shown that egg yolk cholesterol concentration can be reduced by supplementing with antioxidant vitamins (*p *< 0.01) [13]. Supplementation of 1.5% to 3% green and brown marine macroalgae in the diet resulted in reduced total lipid and cholesterol levels in serum and egg yolk, as well as an increase in antioxidants in quail blood [14]. Flavonoid is one of the antioxidants found in the macroalgae. *E. spinosum* acts as an inhibitor of hydroxymethylglutaryl-coenzyme A (HMG-CoA) reductase and acyl-coenzyme A. The level of flavonoids in *E. spinosum* is high with 103.33 mg quersetin/gm [15]. By inhibiting HMG-CoA reductase, the synthesis of very-LDLs (VLDLs) is reduced, leading to less cholesterol transport into egg yolk.

The study found that supplementing pregnant rats with quail eggs resulted in a significant increase in body weight during pregnancy compared to those who did not receive the supplementation. The increase in weight was due to high levels of protein, cholesterol, and fat in quail eggs (Table 2). Conversely, the rats that were not fed with quail egg supplements and had limited food intake for 15-h had the lowest body weight (T0). Previous research studies have shown that a high-cholesterol and high-fat diet, such as beef brain, can increase the weight gain of pregnant rats [16]. The finding of this study indicates the importance of the diet of pregnant rats in determining their body weight during pregnancy. However, the study found that the weight gain of mother rats during pregnancy had no significant effect on the birth weight of their offspring. The birth weight of rat pups born to mothers who received quail egg supplements was not significantly different from those born to mothers who did not receive any supplementation (control group). Previous studies have shown that rat mothers fed with a high-cholesterol diet had offspring with low birth weight, small body size at birth, and slow bone ossification [17]. Other studies have reported that feeding mother rats a high-fat diet generally has no effect on the birth weight of rat pups [18]. However, providing a diet containing 45% fat has been shown to result in offspring with lower birth weights [19]. Therefore, the birth weight of rat pups is influenced by the diet their mother receives during pregnancy.

The condition of blood cells has the potential to be a useful indicator for the detection of metabolic disorders, diseases, structural damage to organ function, the influence of agents or drugs, and stress. Blood cell examination can be a valuable tool for diagnosing diseases and monitoring metabolic disorders. In addition, the blood cell status can be useful for assessing health conditions during pregnancy. A study found that giving quail egg supplements did not significantly increase the levels of RBC, MCV, MCH, MCHC, WBC, LYM, NEUT, RDW, PDW, and MPV compared to the control treatment but were still within normal limits [20]. RBCs play an essential role in transporting Hb, which transports oxygen from the lungs to the tissues. The MCV value, which can indicate the type of anemia based on the size of the erythrocytes, can be used to evaluate the condition of blood cells. A low MCV value indicates that the erythrocytes are small (microcytic), which can be caused by iron deficiency. Furthermore, the MCH value describes the Hb concentration in one erythrocyte cell and can be used to diagnose the type of anemia. The MCHC value is the Hb concentration in a volume of erythrocytes. Both MCH and MCHC values can reflect the health of Hb in the blood. Low MCH and MCHC values indicate low average Hb levels in erythrocytes (hypochromic) so that the color becomes faded.

The administration of quail egg supplement showed significantly different results in the Hb and HCT levels compared to the control treatment. Hb in the control group (T0) was below normal levels, indicating iron deficiency anemia in the pregnant rats. Normal Hb levels in pregnant Sprague Dawley rats at 21 days of gestation are (11.50 gm/dl) [20]. Hb is a strong indicator of iron deficiency anemia. This was further confirmed by the HCT levels, which were below normal levels of 29.18 ± 0.59% (Table 4). Low HCT levels are an indicator of anemia. The normal level of HCT in pregnant mice aged 21 days of gestation is 33.8% [20]. Anemia is a condition characterized by a decrease in RBC mass, indicated by low levels of Hb, HCT, and RBC count. The occurrence of anemia is thought to be caused by the increased nutritional requirements during pregnancy, especially the need for iron. The iron requirement for Sprague Dawley rats during pregnancy is 80 ppm [21]. To increase the iron content in quail eggs, the diet of quail can be supplemented with additional marine macroalgae *E. spinosum* at various concentrations of 3%, 4%, or 5%. The results of the supplementation of *E. spinosum* increase the iron content in the quail eggs (Table 1). Since iron is a crucial component in Hb synthesis.

To increase the iron content in quail eggs, the diet of quail can be supplemented with additional marine macroalgae *E. spinosum* at various concentrations of 3%, 4%, or 5%. The results of the supplementation of *E. spinosum* increase the iron content in the quail eggs (Table 1). Since iron is an essential component in Hb synthesis, adequate iron intake during pregnancy can lead to higher levels of serum Hb and HCT. Moreover, iron is crucial for various physiological processes such as energy production, oxygen transport, oxidation-reduction reactions, myocyte function, and cell division.

The supplementation of quail eggs to pregnant rats has had the highest serum cholesterol levels of 134.00 ± 6.36 (Table 4). The high level of serum cholesterol in treatment T1 was due to the quail egg produced by quail fed commercial feed; the eggs contained a high cholesterol level of 854.97 (mg/100 gm) (Table 1). Consequently, the intake of such eggs increased serum cholesterol levels [22]. However, the quail egg produced by quail fed additional marine macroalgae *E. spinosum* had a lower egg cholesterol content, which did not have a significant effect on increasing serum cholesterol levels in pregnant rats. Moreover, treatment T1 showed the highest LDL level value of 95.57 ± 2.70 (Table 4), plausibly due to the intake of a quail egg supplement diet, which has high cholesterol levels. The results of this study align with previous research, indicating that a high-cholesterol diet given to pregnant rats can increase total serum cholesterol and a tendency for high LDL, as compared to not being given a cholesterol diet [22].

During pregnancy, lipids play a crucial role in cholesterol metabolism. Lipogenesis increases at the onset of the second trimester, while lipolysis is heightened towards the end of the first trimester. Hormonal changes, specifically an increase in estrogen and progesterone, which trigger pancreatic cell hyperplasia in the early second trimester, leading to heightened insulin production and sensitivity. These hormonal changes promote maternal hyperphagia and boost the activity of lipoprotein lipase, thereby elevating fat synthesis and adiposity hypertrophy. Consistent with previous research, pregnant rats exhibited an increase in body weight, particularly those fed a high-fat diet [23]. The weekly weight measurements of the rats demonstrated an increase in their body weight, as presented in Table 2.

The current study proposes that the elevated serum LDL levels in treatment T1 might be related to the high cholesterol content contained in the quail egg supplements. This study is aligned with previous research that has reported an increase in serum cholesterol levels with the administration of cow brains to pregnant rats [16]. Furthermore, other research reveals that a high-fat diet provided to pregnant rats can lead to higher plasma cholesterol concentrations at 20 days of gestation compared to control treatment [24]. High cholesterol consumption might increase the synthesis of VLDL by raising intracellular cholesterol levels in the liver. Both of these mechanisms could contribute to the elevation of LDL levels in the bloodstream [16].

The T4 treatment showed the highest levels of HDL, which is believed to be attributed to the relatively lower cholesterol content present in the quail eggs consumed in the T4 treatment compared to the other treatments listed in Table 1. The results of this study align with previous research that the consumption of low-cholesterol eggs can increase serum HDL levels [25]. Moreover, several research studies have reported that the addition of cholesterol from eggs during weight maintenance conditions can result in an elevation of HDL levels. High intake of cholesterol can also lead to increased LDL and total cholesterol levels, which is not beneficial for overall health. LDL carries cholesterol into tissues through the blood, while HDL facilitates the transport of excessive LDL back to the liver to be synthesized into bile acids and excreted in the feces. Elevated triglyceride levels can cause HDL to undergo changes that result in its breakdown and lower HDL levels. Low HDL levels and high LDL levels are believed to be caused by high cholesterol levels in quail eggs, which can lead to a decrease in levels of apolipoprotein A1, a precursor to HDL formation, and an increase in LDL levels due to an imbalance in HDL production with cholesterol entering the body. In contrast, high HDL levels can help to reduce LDL levels, thereby reducing the risk of physiological disorders.

The supplementation of quail eggs to pregnant rats can lead to the elevation in the serum glucose levels due to the high cholesterol content of the eggs (Table 1). The findings from prior research indicate a significant connection between higher cholesterol levels, decreased serum HDL levels, and increased blood glucose levels. According to previous research, a significant rise in blood glucose levels is observed in pregnant women who consume a diet rich in cholesterol [26]. Furthermore, an increase in both cholesterol and triglyceride levels has a significantly negative impact on the increase of blood glucose levels. Recent studies have indicated that HDL levels may exert a direct influence on glucose metabolism [27].

This study resulted in increased cholesterol and LDL levels in both the T1 and T2 treatments, which could have led to an increase in glucose levels. While the precise underlying mechanism of the influence of dietary cholesterol on blood glucose metabolism has yet to be fully understood, previous studies stated that plasma lipids play a significant role in maintaining blood glucose homeostasis [28]. According to previous studies, a rise in plasma cholesterol levels could activate the flow of pro-inflammatory signals, and excessive cholesterol intake could escalate amyloid A levels, indicative of inflammation. An elevated intake of cholesterol is likely to cause an increase in the concentration of cholesterol in the bloodstream. This, in turn, can lead to inflammation and eventually result in insulin resistance. Other studies have found that cholesterol accumulation could impede pancreatic β-cell function, which could interfere with insulin production. Pancreatic β-cells are responsible for regulating glucose absorption from the bloodstream to the liver, fat, and skeletal muscle cells, as well as producing and releasing insulin.

The study results indicated no significant changes in the levels of MDA and SOD between the control group and the T1, T2, T3, and T4 treatments. However, it was observed that the average level of MDA in the control group tended to be higher than in the other treatments. This result could be related to the 15-h food restriction and higher nutritional requirements during pregnancy compared to normal conditions. This finding is consistent with prior research that suggests an increase in the production of free radicals and lipid peroxides during pregnancy, particularly towards the end of the pregnancy when compared to non-pregnant individuals. The results also indicated that various factors, such as activity, high-fat diet, high-cholesterol diet, and protein malnutrition, could contribute to an increase in MDA levels [29].

The body has a complex network of antioxidant defenses that work to counteract free radicals. Antioxidants, such as SOD, catalase, and glutathione peroxidase, act as the primary defense against free radicals and are essential in protecting against oxidative stress. SOD, a detoxification enzyme present in cells, is among the many antioxidants that support the human body’s defenses against oxidative stress. Oxidative stress is a result of an imbalance between the number of free radicals and endogenous antioxidants. This stress can cause unstable molecules and uncontrolled lipid peroxidation, along with reactive oxygen species. This ultimately results in excessive lipid peroxidation. As a result, levels of MDA, a byproduct of lipid peroxidation, increase. Therefore, MDA is considered a biological marker of oxidative stress [30].

## Conclusion

The result of this study revealed that the provision of marine macroalgae *E. spinosum* with concentrations of 3%, 4%, and 5% can significantly increase the iron content and linearly reduce the cholesterol levels in the quail eggs. Moreover, the supplementation of quail eggs produced by quails fed with marine macroalgae *E. spinosum* with various concentrations of 3%, 4%, and 5% can significantly improve the body weight, Hb levels, and HCT in pregnant rats. Furthermore, the supplementation of quail eggs produced by quail fed with concentrations of 4% and 5% can effectively reduce the cholesterol, LDL, and glucose levels while increasing the serum HDL levels in pregnant rats. These results indicate that quail egg supplements enriched with marine macroalgae *E. spinosum* can serve as a potential source of nutrition for Sprague Dawley rats during the pregnancy period. Further studies are necessary to validate these results and to explore the potential of the marine macroalgae *E. spinosum *in enhancing the nutritional quality of other food products.

## List of abbreviations

Hb, hemoglobin; HCT, hematocrit; HDL, high-density lipoprotein; LDL, low-density lipoprotein; LYM, lymphocytes; MCH, mean corpuscular Hb; MCHC, mean corpuscular Hb concentration; MCV, mean corpuscular volume; MDA, *malondialdehyde*; MPV, mean platelet volume; NEUT*, neutrophils; *PDW, platelet distribution width; RBC, red blood cell; RDW, RBC distribution width; SOD, *superoxide dismutase*; VLDL, very low-density lipoprotein; VTG, vitellogenin.

## References

[1] U.S. Department of Agriculture and U.S. Department of, Health and Human Services (2020). Dietary guidelines for Americans, 2020-2025.

[2] Jouanne M, Oddoux S, Noël A, Voisin-Chiret AS (2021). Nutrient requirements during pregnancy and lactation. Nutrients.

[3] Abd Rahman R, Idris IB, Isa ZM, Rahman RA, Mahdy ZA (2022). The prevalence and risk factors of iron deficiency anemia among pregnant women in Malaysia: a systematic review. Front Nutr.

[4] Garzon S, Cacciato PM, Certelli C, Salvaggio C, Magliarditi M, Rizzo G (2020). Iron deficiency anemia in pregnancy: novel approaches for an old problem. Oman Med J.

[5] Rendón U, Carrillo S, Arellano LG, Casas MM, Pérez F, Avila E (2003). Chemical composition of the residue of alginates (*Macrocystis pyrifera*) extractio. Cub J Agric Sci.

[6] Bess F, Vieira SL, Favero A, Cruz RA, Nascimento PC (2012). Dietary iron effects on broiler breeder performance and egg iron contents. Anim Feed Sci Technol.

[7] Sanjaya R, Sagita YD, Janet SK (2023). Effect of egg on hemoglobin level of pregnant women. J Sci n.a.

[8] Sharma R, Jaitawat A, Jain N, Kantwa SM (2015). Effect of regular egg consumption during pregnancy and lactation on morphological characters in Swiss albino mice. Int J Res Pharm Sci.

[9] Isobe K, Sato N, Ono K, Fujita H, Kawamura M, Sasaki H (2020). Are mice born normally with an egg-only diet?. Dev Biol.

[10] Binev R (2023). Investigations on some indicators in the blood of cattle with orosthenic activity tongue rolling. J Adv Vet Anim Res.

[11] Apriyanto YS, Iriyanti N, Tugiyanti E (2021). The effect of supplementation of avocado seed flour (*Persea americana**Mill*) in feed on blood lipids profile and egg yolk cholesterol of Japanese Quail (*Corturnix-corturnix japonica*). Anim Prod.

[12] Xie C, Elwan HAM, Elnesr SS, Dong XY, Zou XT (2019). Effect of iron glycine chelate supplementation on egg quality and egg iron enrichment in laying hens. Poult Sci.

[13] Mohiti-Asli M, Zaghari M (2010). Does dietary vitamin E or C decrease egg yolk cholesterol?. Biol Trace Elem Res.

[14] Abu Hafsa S (2019). Effects of dietary supplementation with green and brown seaweeds on laying performance, egg quality, and blood lipid profile and antioxidant capacity in laying Japanese quail. Egypt Poult Sci J.

[15] Rismayanti NLPM, Husni A (2021). Antioxidant activity of methanolic extract of Eucheuma spinosum extracted using a microwave. IOP Conf Ser: Earth Environ Sci.

[16] Desmawati D, Mahdiyah AY, Kadri H (2023). Effect of high fat and cholesterol diet on total blood cholesterol levels in pregnant Wistar rats. Maj Kedokt Bdg.

[17] Mangu SR, Patel K, Sukhdeo SV, Savitha MR, Sharan K (2022). Maternal high-cholesterol diet negatively programs offspring bone development and downregulates hedgehog signaling in osteoblasts. J Bio Chem.

[18] Desai M, Jellyman JK, Han G, Beall M, Lane RH, Ross MG (2014). Maternal obesity and high-fat diet program offspring metabolic syndrome. Americ J Obstet Gynec.

[19] Dodson RB, Miller TA, Powers K, Yang Y, Yu B, Albertine KH (2017). Intrauterine growth restriction influences vascular remodeling and stiffening in the weanling rat more than sex or diet. AJP Heart Circ Phys.

[20] Kim J-C, Yun H-I, Lim K-H, Suh JE, Chung M-K (2000). Haematological values during normal pregnancy in Sprague-Dawley rats. Comp Haematol Int.

[21] Lee JK, Ha J-H, Collins JF (2021). Dietary iron intake in excess of requirements impairs intestinal copper absorption in Sprague Dawley rat Dams, causing copper deficiency in suckling pups. Biomed.

[22] Sáez T, Pageé A, Kirschenman R, Quon A, Spaans F, Davidge ST (2023). A high cholesterol diet during late pregnancy impairs long-term maternal vascular function in mice. Arterioscler Thromb Vasc Biol.

[23] Desai M, Ferrini MG, Han G, Narwani K, Ross MG (2020). Maternal high fat diet programs male mice offspring hyperphagia and obesity: mechanism of increased appetite neurons via altered neurogenic factors and nutrient sensor AMPK. Nutrients.

[24] Cerf M, Herrera E (2016). High fat diet administration during specific periods of pregnancy alters maternal fatty acid profiles in the near-term rat. Nutrients.

[25] Shakoor H, Khan MI, Sahar A, Khan MKI, Faiz F, Basheer Ahmad H (2020). Development of omega-3 rich eggs through dietary flaxseed and bio-evaluation in metabolic syndrome. Food Sci Nutr.

[26] Zhang Y, Lan X, Li F, Sun H, Zhang J, Li R (2022). Dietary cholesterol and egg intake are associated with the risk of gestational diabetes: a prospective study from Southwest China. BMC Pregnancy Childbirth.

[27] Siebel AL, Heywood SE, Kingwell BA (2015). HDL and glucose metabolism: current evidence and therapeutic potential. Front Pharmacol.

[28] Zhang Y, Lan X, Cai C, Li R, Gao Y, Yang L (2021). Associations between maternal lipid profiles and pregnancy complications: a prospective population-based study. Am J Perinatol.

[29] Sinha S, Patro N, Tiwari PK, Patro IK (2020). Maternal spirulina supplementation during pregnancy and lactation partially prevents oxidative stress, glial activation and neuronal damage in protein malnourished F1 progeny. Neurochem Int.

[30] Cordiano R, Di Gioacchino M, Mangifesta R, Panzera C, Gangemi S, Minciullo PL (2023). Malondialdehyde as a potential oxidative stress marker for allergy-oriented diseases: an update. Molecules.

